# Prevalence of Pulmonary Tuberculosis and Associated Factors Among HIV Positive Patients Attending Antiretroviral Therapy Clinic at Arba Minch General Hospital, Southern Ethiopia

**DOI:** 10.2174/1874285801812010163

**Published:** 2018-05-31

**Authors:** Mohammedaman Mama, Aseer Manilal, Haile Tesfa, Hawa Mohammed, Endeshaw Erbo

**Affiliations:** Department of Medical Laboratory Science, College of Medicine and Health Sciences, Arba Minch University, Arba Minch, Ethiopia

**Keywords:** Prevalence, Pulmonary tuberculosis, HIV positive patients, Acid fast staining, Statistical package, Mobilization

## Abstract

**Background:**

Tuberculosis (TB) is an extremely contagious disease detrimentally affecting virtually every organ, most importantly the lungs. Pulmonary complications have been one of the commonest causes of morbidity and mortality since the advent of AIDS (Acquired Immune Deficiency Syndrome) pandemic. The AIDS virus has considerably reshape the epidemiology of TB by widening the risk of reactivating latent TB, increasing the possibility of TB infection once contracted to tubercle bacilli (re-infection) and by elevating the risk of rapid progression instantly after the infection. In this background, this study is intended to understand the prevalence of pulmonary tuberculosis and associated factors amongst Human Immunodeficiency Virus (HIV) positive patients attending antiretroviral therapy (ART) clinic in Arba Minch General hospital during the study period (March to May, 2016).

**Methods:**

A cross-sectional study was carried out at Arba Minch Hospital from March to May, 2016. To assess the associated factors, a pre-tested structured questionnaire has been used. Sputum samples were collected and examined microscopically by using acid fast staining. The data was analyzed using Statistical Package for Social Services, version 20.

**Results:**

Totally, 291 HIV positive patients were included in this study of which 71.5% were females and 28.5% were males. It was found that 42.3% of respondents were in the age ranged between 31-40 years. Of the 291 patients screened, 21 were positively diagnosed with pulmonary TB making the overall prevalence rate of 7.2%. From this study, it was revealed that CD4 count, previous history of tuberculosis and smoking were the significant predictors of tuberculosis (p˂0.05) in HIV patients.

**Conclusion:**

The results of the present study envisaged that the prevalence of HIV/TB co-infection was 7.2%. Previous history of TB, CD4 count less than 200/μl, and smoking habit were the possible risk factors elucidated. Therefore, TB screening among HIV-positive patients, public awareness, and community mobilization should be encouraged.

## 1 INTRODUCTION

Tuberculosis (TB) is a dreadful bacterial disease caused by *Mycobacterium tuberculosis (M. tuberculosis*), which mostly affects the lungs (pulmonary TB), besides it can affect other organs as well (extra-pulmonary TB) [1]. It is documented that 9.6 million people contracted with TB and 1.5 million deaths occurred in 2014 [2]. In fact, TB is the most common opportunistic disease which kills those infected with Human Immunodeficiency Virus (HIV). Recently, WHO reported that 12% of the 9.6 million new TB cases were HIV-positive [2]. It is known that in HIV patients, active TB is occurred by the reactivation of endogenous latent disease as well as re-infection with a new strain [3]. Epidemiological studies evinced that, co-infection with HIV can elevate the risk of latent TB reactivation by 20-fold and is the most potent risk factor known for the advancement of *M. tuberculosis* infection to active disease [4]. This further led to the parallel pandemic of tuberculosis in some sub-Saharan African populations where 10%–15% of the adult population suffered from multiple infections. In 2014, 0.4 million deaths that occurred as a result of TB were in HIV-infected individuals [2]. Recently, the WHO reported that TB ranks alongside HIV as the lethal infectious disease across the world. Both the diseases were recognized as an “effectively joint top killers” responsible for an approximate 1.5 million deaths in 2014 [2].

TB continuous as an enormous public health issue amongst the developing countries, due to HIV pandemic, poverty, movement of displaced people and emergence of multidrug-resistant strains [5]. An erstwhile study evidenced that, in most of the developing countries, HIV pandemics, diabetes, malnutrition, alcoholism, smoking cigarette, active TB contact, extreme poverty, and homelessness are common identified risk factors pertaining to tuberculosis [6]. TB has been implicated as a significant cause of morbidity and mortality in Ethiopia. For instance, Ethiopia is enlisted seventh amongst in the countries with maximum TB burden in the world [7]. The prevalence of TB among HIV positive patients in Ethiopia currently stands at 9.1% [8]. In the year 2014, it was identified around 200,000 new TB cases in Ethiopia [2] and exacts an enormous toll of 37,500 in Ethiopian population [9]. Besides, it is one of the five most affected countries in Africa with HIV [2]. Ethiopia is a developing country, where economic imbalances are quite rampant, literacy is low and basic health service delivery is scarce; determining the prevalence of tuberculosis among HIV patients is critical to prevent opportunistic infections like tuberculosis. Moreover, the Ethiopian Government has prioritized TB control as one of the major health program packages in the country’s Health Sector Development Program. A perusal of the literature indicated that there is a dearth of the studies pertaining to the prevalence of TB amid HIV patients in Arba Minch province of Ethiopia. For this reason, the present study is initiated to determine the prevalence of pulmonary tuberculosis and associated factors among HIV patients attending ART clinic at Arba Minch General Hospital, South Ethiopia.

## 2 MATERIALS AND METHODS

### 2.1 Study Design, Period and Description of the Study Area

A cross sectional facility based study was designed and performed in the ART clinic of Arba Minch Hospital from March to May 2016. Arba Minch is located in the Gamo Gofa Zone of the Southern Nations, Nationalities, and Peoples Region (SNNPR) around 505 kilometers South of Addis Ababa. The ethical clearance of the study protocol was accorded from the Research Committee of College of Medicine and Health Sciences, Arba Minch University.

### 2.2 Study Participants

#### 2.2.1 Sample size Determination and Sampling Technique

The sample size was computed using a sample size determination formula for the estimation of the single population proportion. In the present study, 22% prevalence of TB among HIV cases opted from a previous report [10]. After considering 95% of the confidence interval and 5% of marginal error, the initial sample size was 264. Finally, by assuming 10% of non –response rate, the final sample size was consolidated as 291. The respondents were chosen using systematic random sampling technique, by taking an average of 70 HIV patients consulting daily at ART clinic. Systematic random sampling technique was opted. The *K*^th^ value was calculated and subjects were preferred by lottery method.

### 2.3 Data Collection, Sampling Procedure, and Sample Processing

Data pertained to socio-demographic characteristics, and other associated factors of the patients were obtained by face to face interview method using semi-structured questionnaire. The recent CD4 counts of the respective patients were procured from the medical record. The expectorated sputa were collected three times (spot -morning-spot) in sterile leak-proof plastic containers. Samples were decontaminated with sodium hypochlorite and concentrated by centrifugation subsequently freeing the bacteria ready for staining. Smears were prepared, fixed and stained by Ziehl-Neelsen staining methods as described elsewhere [11]. The smears were then carefully inspected using the zoom stereo microscope to confirm the presence of acid-fast bacilli. The case was defined as positive when two or more smears positive for acid-fast staining.

### 2.4 Data Quality

A semi-structured questionnaire was used to obtain the data. The questionnaire was pretested on 10%, who were not included in the study subject. The quality of Ziehl-Neelsen staining method was inspected by preparing the daily reagents with the standard reagent preparation methods.

### 2.5 Data Analysis

The data was entered onto software Epi-data version 13.1. and cross-checked and data cleaning was performed. Afterward, the data was entered into Statistical Package for Social Sciences (SPSS) program version 20.0 for analysis. Odds ratio (OR) and 95% confidence intervals (95% CI) was calculated for each predictor variable. The P-value of ≤ 0.05 was considered statistically significant.

## 3 RESULTS

### 3.1 Socio-Demographic Status

A total of 291 HIV positive patients were included, of which 71.5% (*n*=208) of the patients were females and 28.5% (*n*=83) were males Table (**1**). It was found that 42.3% (*n*=223) of respondents were in age ranged between 31-40 years and the rest (*n*=79; 27.1%) were in the age ranged between 41-50 years. The average age of respondents of this study was 37.43 years (mean S.D. = 9.21). Concerning the marital status of the study population, 53% (*n*=154) of the respondents were married and 21.6% (*n*=63) were divorced. Majority (*n*= 194; 66.7%) of the respondents are living in urban and 33.3% (*n*=97) are living in rural areas.

### 3.2 Prevalence and Risk Factors

Of the 291 subjects screened, 21 were positively diagnosed with pulmonary TB Figs. (**1** and **3**) giving the overall prevalence rate of 7.2% Fig. (**2**). In the present study, more than a half (*n*=158; 54.3%) of participants had CD4 count ranged between 200-500/μl and 21.6% (*n*=63) of respondents had CD4 count <200/μl. In addition, the study also showed that 29.2% (*n*=85) and 3.7% (*n*=11) of the respondents had a history of TB and smoking habit respectively. During the study, 97.3% (n=283) respondents were on ART. It has been identified that majority of TB/HIV co-infected persons were in the age group ranged between 31-50 years. Female patients had a higher rate of prevalence (27.5% *vs.* 1% in men). Of the 21 TB/HIV cases, 4.1% (*n*=12) were newly diagnosed cases while 3.1% (*n*=9) had a history of TB treatment (relapse) and had strong association that is 6 times more vulnerable to develop TB. (AOR=6.13 [1.7-3.05]). HIV cases whose CD4 count less than 200 were more than nine times likely to develop tuberculosis compared to those whose CD4 count greater than 500 (AOR= 9.04[4.23- 79.11]), besides, a significant statistical relationship was found between smokers and nonsmokers (AOR=4.019[0.01-0.16]) (Tables **2** and Table **3**).

## 4 DISCUSSION

Albeit the recent WHO Tuberculosis report evidenced that prevalence of tuberculosis has been declining globally, we are far away from achieving TB free world even in 2050 [12]. In Ethiopia, overall prevalence of TB is 261 per 100, 1000 and is the most common opportunistic infection in patients with HIV/AIDS [13]. Further, people living with HIV are most vulnerable to contracting active TB because of the deficiency of immune response. It is envisaged that TB can affect young people in a disproportionate manner [9]. Regarding the demographic variables, it was found that the majority (*n*=223) of respondents were in age ranged between 31-40 years. A recent national survey report revealed that 58% of prevalent TB cases in Ethiopia are less than 35 years of age [14].

In the present study, females have outnumbered males, of the 291 HIV positive patients participated, 71.5% (*n*=208) were females and 28.5% (*n*=83) were males. Our results are in accordance with the previous study conducted at North West part of Ethiopia. As noted by the authors, majority of the study participants were females (64.2%) [15]. The possible reason for high prevalence of TB in females might appertain to their biological factor, which makes them more vulnerable to HIV infection.

The present study evidenced that the prevalence of TB-HIV co-infection was 7.2%. Our findings are in accordance with previous studies conducted on international and national geographical locales such as Northern Tanzania (8.5%) [16], Nigeria (9.6%) [17] and other regions of Ethiopia such as Bahir Dar (10.1%) [5] and Southwest Ethiopia (8.1%) [18]. In contrast, relatively higher prevalence has been reported from other countries such as Nepal (27.3%) [19] and Nigeria (34.5%) [20]. This divergence in the results could be due to the differences in inclusion and exclusion criteria. As patients already diagnosed with tuberculosis, extrapulmonary tuberculosis and started tuberculosis treatments were turf out in our study, this could be lower than the rate reported by other studies. Howbeit, as compared to our study, a lower rate of prevalence was reported from South Africa (3.8%) [21]. This variation could be due to divergences of HIV infection rate in the population, availability of TB- diagnostic facilities, health care awareness of the community. It is documented that in sub-Saharan Africa, the frequency of HIV/TB co-infection is very high (50%) [22]. In the developing countries like Ethiopia, TB is one of the most common debilitating diseases among the patients living with HIV/AIDS. The rates of HIV/TB co-infection have been reported to vary in different regions of Ethiopia. The routine data from health facilities in Ethiopia indicated that the prevalence rate was 31% [23]. Howbeit, the above-cited studies corroborated all forms of tuberculosis, while in our study, pulmonary tuberculosis was only included.

In the present study, of the total TB cases, 42.9% of tuberculosis infection occurred in low CD4 level (<200/μl). An earlier study conducted in Ethiopia adduced that about two third (67.2%) of the patients had CD4 count less than 200/μl [24]. Likewise, a study done in India, opportunistic infections pertaining to the CD4 level among HIV seropositive patients revealed that 17% of prevalence of tuberculosis was observed as soon as the CD4 level reduced below 200/μl [25]. In the present study, it was observed that the patients with a CD4 count below 200/μl have an association with TB infection (AOR=9.04 [4.23- 79.11]) compared to patients who had a CD4 count greater than 500/μl. Past studies in Ethiopia and India have pointed to the fact that CD4+ T cell count is lower among co-infected patients as compared to HIV infected alone and severe immune suppression has been observed in those with CD4+ T cell count <200 cells/μl [15, 25].

In the present study, majority of the participants manifested no typical symptoms of tuberculosis. This could be due to the failure to develop characteristic granuloma of immunogenic origin [26]. Seventeen patients with tuberculosis were on antiretroviral drugs and yet developed tuberculosis; this evidenced that there is an urgent need to do regular and prompt tuberculosis screening by sputum microscopy to diagnose tuberculosis in HIV patients on treatment. Four patients with tuberculosis were not yet eligible for antiretroviral drugs. If tuberculosis is diagnosed in these patients and correctly staged, they would be qualified for beginning antiretroviral drugs and Cotrimoxazole Prophylaxis (CP) [27].

It is a proven fact that tobacco smoking and alcohol consumption reduces the immune response in human and increases the risk for TB [28, 29]. In this study, we are unable to find the prevalence of TB statistically different in alcoholic and non alcoholic patients. However, the prevalence of TB was considerably higher in chain smokers as compared with non-smokers (P <0.05).

### 5 LIMITATIONS OF THE STUDY

The present study was a hospital-based analysis of the HIV/TB co-infected patients and the findings might not be generalizable. Since we used only smear microscopy of AFB staining, not the chest X-ray as a supportive diagnostic tool for all the HIV infected persons, we might have missed some cases of tuberculosis. In addition, the cultural technique was not performed because of the limited laboratory facilities.

## CONCLUSION

As a conclusion, the present study envisaged that the prevalence of HIV/TB co-infection was 7.2%. Previous history of TB, CD4 count less than 200/μl, and smoking habit were the possible risk factors elucidated in the present study. Therefore, TB screening among HIV-positive patients, public awareness, and community mobilization should be encouraged. In addition, large-scale studies on the trends in TB/HIV co-infection and associated factors should also be implemented across the country.

## Figures and Tables

**Fig. (1):**
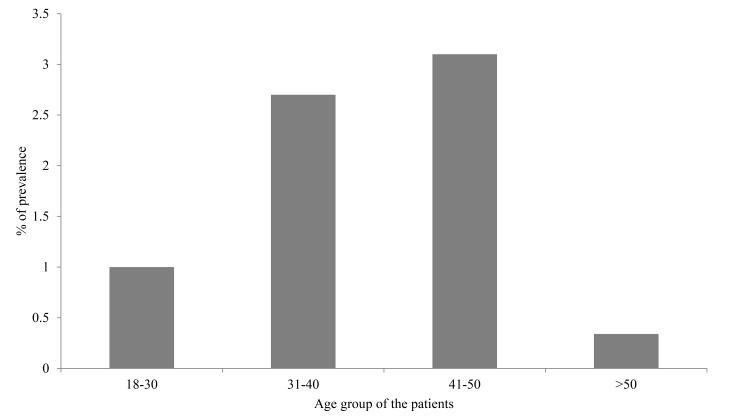
The prevalence of PTB in different age group of the patients attending ART clinic in Arba Minch General hospital, South Ethiopia from March to May, 2016.

**Fig. (2):**
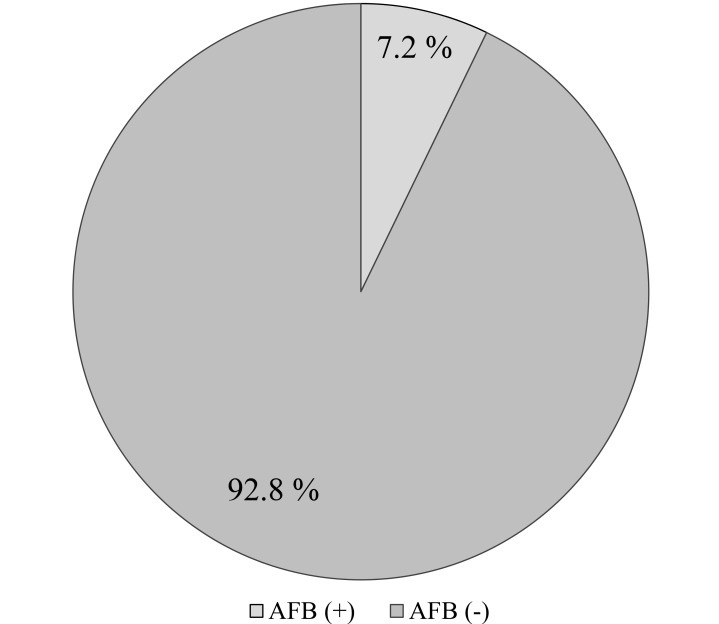
Overall prevalence rate of PTB in HIV patients attending ART clinic in Arba Minch General hospital, South Ethiopia from March to May, 2016.

**Fig. (3):**
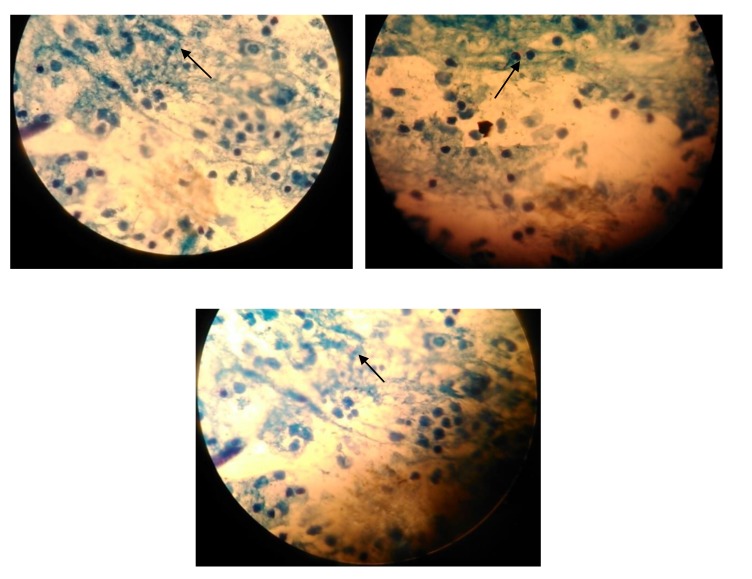
Acid fast staining of positive samples sourced from HIV positive patients attending ART clinic in Arba Minch General hospital, South Ethiopia from March to May, 2016.

**Table 1:** Socio demographic characteristics of HIV patients attending ART clinic in Arba Minch General hospital, South Ethiopia during the study period (March to May, 2016).

**Characteristics**		**Frequency**	**Percent**
**Age**	18-30	62	21.3
	31-40	123	42.3
	41-50	79	27.1
	˃50	27	9.3
**Sex**	Male	83	28.5
	Female	208	71.5
**Marital Status**	Married	154	53
	*Single*	58	20
	Divorced	63	21.6
	Widow/Widower	16	5.5
**Occupational Status**	Employed	32	12.3
	Unemployed	42	14.4
	Merchant	75	25.8
	House wife	97	33.3
	Farmer	45	15.5
**Educational Status**	Illiterate	123	42.3
	Primary	87	29.9
	Secondary	46	15.8
	College/University	35	12
**Residence**	Urban	194	66.7
	Rural	97	33.3
**Family Size**	1-5	166	56.7
	>5	125	43
**Monthly Income**	Less than 1000	245	84.2
	1000-2000	31	10.7
	>2000	15	5.1

**Table 2:** Associated factors of tuberculosis among HIV positive patients in Arba Minch General hospital, South Ethiopia during the study period (March to May, 2016).

**Predictors**		**Pulmonary Tuberculosis Status**				
		**Positive**	**Negative**	**COR[CI]**	**AOR[CI]**	**P-value**
		No (%)	No (%)			
Age	18-30	3(1.0)	59(20.3)	1.6[0.25-10.08]	1.2[0.26- 5.50]	0.62
	31-40	8(2.7)	116(39.9)	1.31[0.25-6.86]	0.87[0.179-4.22]	0.75
	41-50	9(3.1)	70(24.1)	0.62[0.13-2.99]	0.76[0.05-12]	0.54
	˃50	1(0.34)	25(8.6)	1.00		
Sex	Male	3(1.0)	80(2.7)	1.00		
	Female	18(6.2)	190(65.3)	1.789[0.58- 5.48]	1.25[0.242- 6.45]	0.30
Smoking	Yes	5(1.7)	8(2.7)	1.00		
	No	16(5.5)	262(90.0)	8.64 [2.59-13.05]	4.019[0.01-0.16])	**0.01**
Alcohol drinking	Yes	9(3.0)	65(22.3)	0.42 [0.17-1.04]	0.49[0.13-1.78]	0.06
	No	12(4.1)	205(70.4)	1.00		
Asthma	Yes	3(1.0)	5(1.7)	1.01[0.12-8.13]	0.199[0.00-4.42]	0.99
	No	18(6.2)	265(91.1)	1.00		
Nutritional Status	Very good	1(0.34)	5(1.7)	1.00		
	Good	1(0.34)	22(7.5)	1.23 [0.52- 2.92]	0.43 [0.27-0.68]	0.92
	Not good	19(6.5)	243(83.5)	3.45[0.185-11.48]	0.45[0.285-9.48]	0.72
Diabetic Mellitus	Yes	1(0.34)	21(7.2)	0.08 [0.03-0.24]	0.02[0.00-0.12]	**0.00**
	No	20(6.8)	249(85.6)	1.00		
Previous History of TB	Yes	9(3.0)	76(26.1)	2.17[0.03-0.75]	6.13 [1.7-3.05]	**0.02**
	No	12(4.1)	194(66.7)	1.00		
Presence of TB in Family	Yes	5(1.7)	26(8.9)	2.34 [2.11-14.80]	1.76[1.10-5.60]	0.070
	No	16(5.4)	244(83.8)	1.00		
History of Respiratory Tract Infections	Yes	6(2.1)	106(36.4)	1.00[0.40-2.50]	0.40[0.10-1.60]	0.99
	No	15(5.2)	16(5.5)	1.00		
HIV Case	On ART	19(6.5)	264(90.7)	1.74 [2.59-13.05]	0.92[0.89-.6]	0.21
	Pre ART	2(0.68)	6(2.1)	1.00		
CD4 Count	<200/μl	9(3.1)	54(18.6)	11.04(4.23- 79.11)	9.04[4.23- 79.11]	0.01
	200-500/μl	10(3.4)	148(50.9)	7.627(0.881-49.859)	2.24[1.01-27.13]	
	>500/μl	2(0.69)	68(23.3)	1.00		

**Table 3:** Clinical symptoms of tuberculosis among HIV positive patients in Arba Minch General hospital, South Ethiopia during the study period (March to May, 2016).

**Symptom**		**Positive**	**Negative**	**CRO[CI]**	**AOR[CI]**	**P-value**
		No (%)	No (%)			
**Cough**	Yes	2(0.69)	47(16.2)	2.10[0.47-9.34]	1.34[0.90-4.04]	0.32
	No	19(6.5)	223(76.6)	1.00		
**Cough length**	1 week	1(0.34)	34(11.7)	0.87(0.179-4.22	0.25[0.34-2.93]	0.45
	≥2 week	1(0.34)	13(4.4)	1.00		
**Fever**	Yes	5(1.7)	93(31.9)	1.00		
	No	16(5.5)	177(60.8)	4.70[0.60-4.81]	1.39[0.87-2.01]	0.31
**Night sweat**	Yes	2(0.69)	29(9.9)	2.14[0.25-5.16]	0.92[0.25-34.08]	0.86
	No	19(6.5)	241(82.8)	1.00		
